# Assessing progress and challenges towards malaria elimination in Kampong Speu, Cambodia: analysis of *Plasmodium vivax* and mixed infections, 2019–2023

**DOI:** 10.5365/wpsar.2025.16.1251

**Published:** 2025-12-24

**Authors:** Kanha Ly, Sophanith Ung, Maria Concepcion Roces, Dysoley Lek, Po Ly

**Affiliations:** a Kampong Speu Provincial Health Department, Kampong Speu, Cambodia.; b South Asia Field Epidemiology and Technology Network, Phnom Penh, Cambodia.; c South Asia Field Epidemiology and Technology Network, Manila, Philippines.; d National Center for Parasitology, Entomology, and Malaria Control, Phnom Penh, Cambodia.

## Abstract

Malaria is a life-threatening but preventable disease caused by *Plasmodium* parasites transmitted through bites of infected female *Anopheles* mosquitoes. According to the World Health Organization, the Western Pacific Region reported 1.7 million malaria cases in 2023, of which *Plasmodium vivax* accounted for 28.9% of cases and approximately 3500 malaria-related deaths. This reflects a decrease in the incidence of malaria cases and associated mortality compared to 2022, highlighting progress but underscoring persistent challenges. Cambodia, with its goal to eliminate malaria by 2025, continues to face public health challenges, particularly from *P. vivax* and mixed-species infections. This report provides an in-depth epidemiological analysis of malaria cases and radical cure treatment outcomes for *P. vivax* and mixed-species infections in Kampong Speu Province from 2019 to 2023. Data were drawn from Cambodia’s national Malaria Information System and radical cure treatment records. The analysis demonstrated a substantial increase in malaria screening, primarily conducted by village malaria workers, while the number of confirmed malaria cases continued to decline. The annual parasite incidence dropped from 23.8 per 1000 at-risk individuals in 2019 to 0.7 per 1000 in 2023. Radical cure treatment completion rates among eligible cases improved from 78% in 2019 to 98% in 2023. Significant progress has been made towards malaria elimination. However, males aged 15–49 years, particularly forest-goers in the Kampong Speu operational district, remain the most at-risk group. In support of malaria elimination, it is recommended to enhance prevention measures, increase screening and ensure 100% radical cure treatment for all eligible cases in high-risk populations.

Malaria is a life-threatening but preventable disease caused by parasites transmitted to humans through the bites of infected female *Anopheles* mosquitoes. Five parasite species cause malaria in humans, with *Plasmodium falciparum* and *Plasmodium vivax* posing the greatest risk. (*1*) Globally, an estimated 263 million malaria cases were reported in 2023, showing a slight decline from 249 million cases in 2022. The number of malaria-related deaths has trended downward from 864 000 in 2000 to 597 000 in 2023. (*1*-*3*) In the World Health Organization (WHO) Western Pacific Region, over 1.7 million cases were recorded in 2023, with *P. vivax* accounting for 28.9% of these cases and approximately 3400 related deaths. This represented an 11% increase in cases and 4% increase in deaths compared to 2020 figures. (*1*)

The Greater Mekong Subregion aims for zero malaria cases by 2030, (*4*) aligning with the regional malaria elimination goal. As part of this initiative, Cambodia has set a national goal to eliminate all species of malaria parasites affecting humans by 2025, as outlined in the *Cambodian National Strategic Plan for Malaria Elimination, 2011–2025*. (*5*) The strategy focuses on universal access to early malaria diagnosis and treatment, and emphasizes the identification and treatment of all malaria cases, particularly among mobile and migrant populations. This strategy includes treating both *P. falciparum* gametocytes and the dormant liver stage of *P. vivax* to prevent relapse and further transmission through radical cure with primaquine. The strategy also includes managing the challenge of glucose-6-phosphate dehydrogenase (G6PD) deficiency, (*5*) which affects  10–19% of Cambodian males and up to 13.8% of females, with regional and ethnic variations. (*6*)

In 2023, Cambodia reported 1384 malaria cases, comprising 94% *P. vivax*, 2% *P. falciparum* and 3% *Plasmodium malariae* and *Plasmodium knowlesi* cases. *P. vivax* cases decreased by 63% and *P. falciparum* cases by 95% compared to 2022. (*7*)

In line with Cambodia’s 2022 national treatment guidelines for malaria, all cases should be treated with a 3-day artesunate–mefloquine regimen as first-line treatment. Patients with *P. vivax* or mixed infections should receive a 14-day course of primaquine (0.25–0.5 mg/kg/day) as a radical cure, provided they are G6PD-normal and weigh at least 20 kg, while low-dose primaquine is given for *P. falciparum* gametocyte clearance (**Fig. 1**). (*8*)

**Fig. 1:**
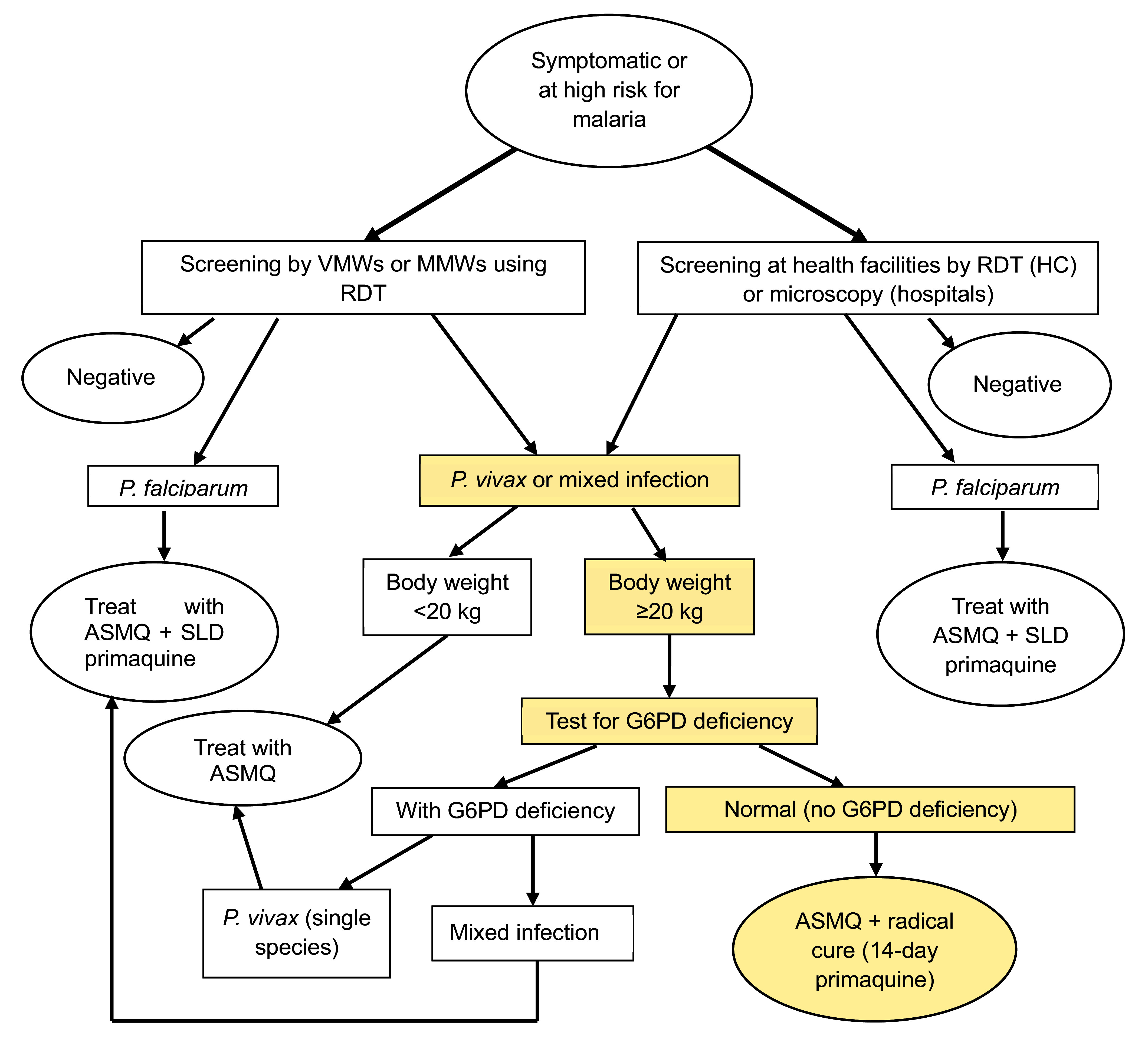
Screening, diagnosis and treatment of non-pregnant malaria cases in Cambodia^a^

[**insert** Figure 1]

Kampong Speu, one of Cambodia’s 25 provinces, lies approximately 48 km west of Phnom Penh, with a population of 978 189 in 2024. (*9*) The province is largely characterized by mountainous and forested areas, with agriculture being a significant industry. It is one of Cambodia’s 21 malaria-endemic provinces. (*5*)

The Kampong Speu Provincial Health Department oversees four operational districts (ODs) and includes a provincial referral hospital, three district referral hospitals, 59 health centres and four health posts (**Fig. 2**). The provincial malaria programme is supported by 296 village malaria workers (VMWs) and 15 mobile malaria workers who operate at the community level.

**Fig. 2:**
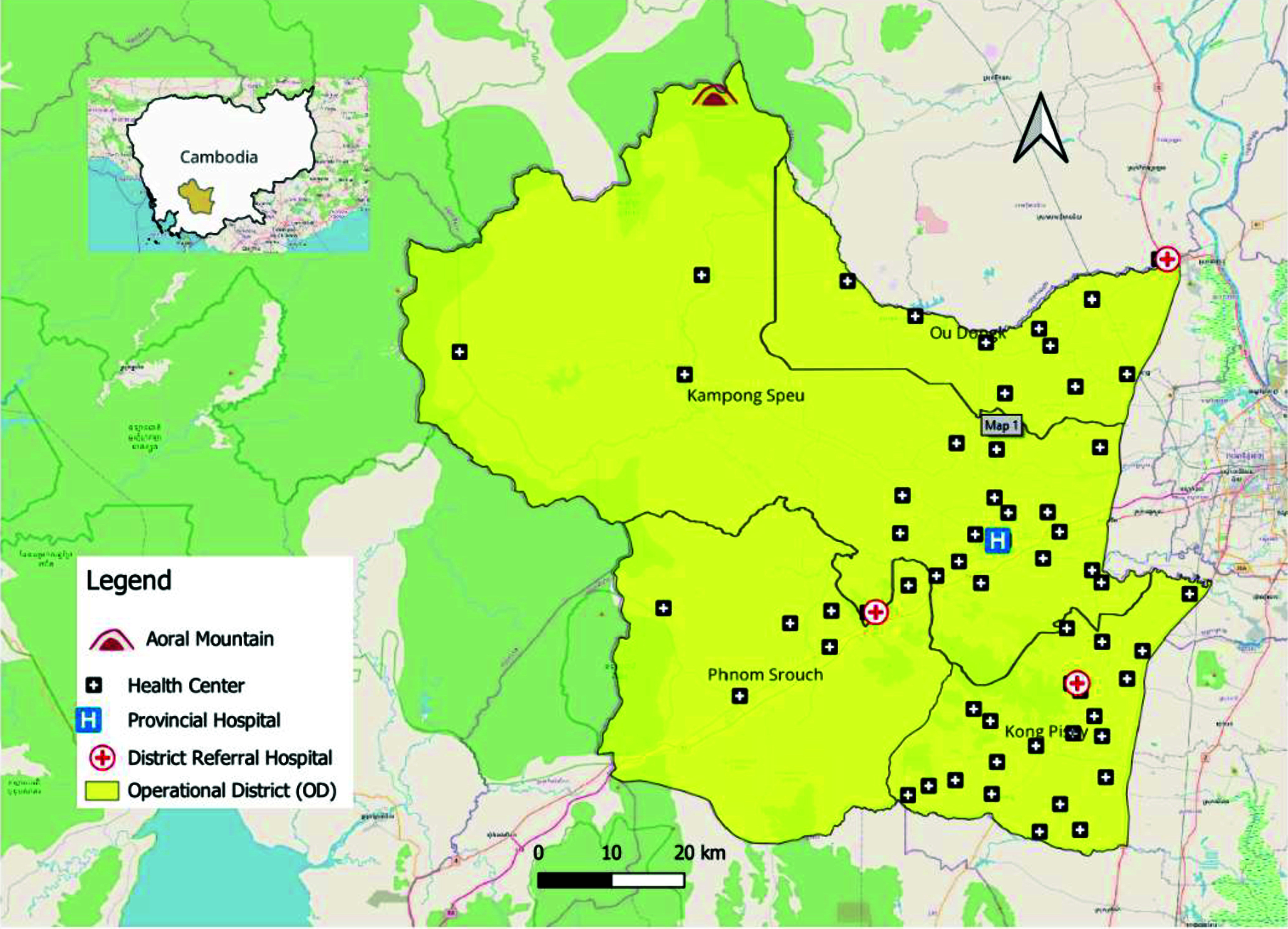
Map of Kampong Speu Province, Cambodia, indicating the location of hospitals and medical facilities in four operational districts, 2019–2023

[**insert** Figure 2]

This report describes the epidemiological characteristics of patients with *P. vivax* or mixed species malaria in Kampong Speu from 2019 to 2023 and their associated treatment outcomes.

## Methods

This study reports on data from malaria cases in Kampong Speu Province, focusing on cases involving *P. vivax* and mixed infections of *P. vivax* and *P. falciparum*. Data were sourced from the Malaria Information System (MIS) and from aggregated monthly radical cure treatment reports for Kampong Speu, covering the years 2019–2023.

### Malaria Information System data

Data from the MIS included comprehensive records collected by VMWs and health facilities (health centres, district and provincial hospitals). The data set includes the patients’ demographic information (age, sex, residential address), body weight and mobility status, the health facility where the patient sought care, the type of test performed, that is, rapid diagnostic test (RDT) and/or microscopy, and corresponding results including malaria species detected (*P. falciparum, P. vivax, P. malariae*, mixed infection), the case notification date, severity classification, treatment administered and the final outcome of the illness.

### Radical cure treatment data

Radical cure treatment data were sourced from each OD in Kampong Speu Province. The data set, provided in Microsoft Excel format (Microsoft Corporation, Redmond, United States of America), aggregated information from VMWs and health facilities about patients treated for *P. vivax* or mixed infections. Key variables included the following:

number of confirmed *P. vivax* or mixed species malaria cases;
number of patients eligible for G6PD testing and associated test results;
number of patients eligible for radical cure based on G6PD test results;
number of patients who started radical cure treatment; and
number of patients who completed the radical cure regimen.

### Data cleaning and anonymization

Before analysis, the data set underwent several preparation steps. Duplicate records were identified using conditional formatting based on name, age, sex and date of notification, and were subsequently removed. Missing values were coded as “99” to enable identification and handling during analysis. The entire data set was reviewed to detect anomalies, such as out-of-range ages, sex mismatches and incorrect or inconsistent treatment records, through automated filters and a manual review. Records with detected anomalies were then verified and corrected using patient registration log books from VMWs and health facilities. Unverified and inconsistent records were excluded from the data set before analysis. Finally, patients’ names were removed following data cleaning to ensure data anonymization and confidentiality.

### Descriptive analysis

Descriptive statistics calculated frequencies and percentages per year, OD, patient demographics, diagnostic test results, treatment received and treatment outcomes.

The annual parasite incidence (API) for Kampong Speu Province and its ODs was determined by dividing the number of *P. vivax* and mixed-infection malaria cases by the at-risk population. The at-risk population was defined as individuals living in: (1) villages with reported malaria cases, indicating confirmed local transmission; and (2) villages located within 5 km of a forest or body of water (for example, a stream). These areas are considered environmentally at risk due to increased exposure to mosquito breeding habitats and higher vector density, even in the absence of confirmed cases.

## Results

### Screening of individuals and test results

**Fig. 3:**
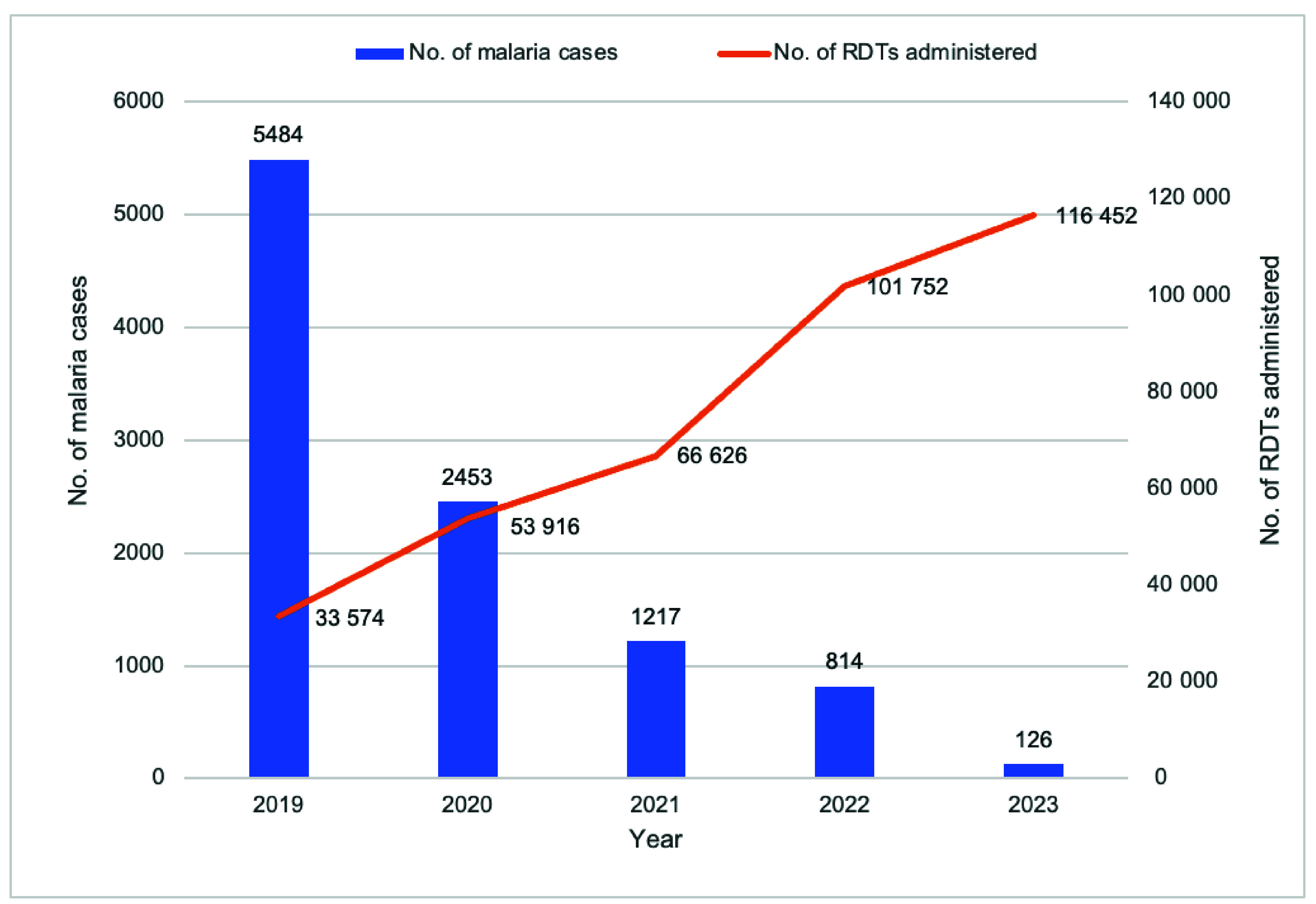
Number of malaria cases identified and number of rapid diagnostic tests administered by year, Kampong Speu Province, Cambodia, 2019–2023

Between 2019 and 2023, the number of individuals tested for malaria saw a significant increase, from 33 574 in 2019 to 116 452 in 2023. Despite a substantial rise in the number of tests conducted, the number of confirmed malaria cases in the province dropped markedly, from 5484 cases in 2019 to 126 cases in 2023 (**Fig. 3**). VMWs played a crucial role in diagnosing malaria cases, identifying 60% (6094/10 094) through RDTs, while the remaining 40% were diagnosed at public health facilities.

[**insert** Figure 3]

### Malaria species identification

**Table 1** presents the type of malaria species identified annually among diagnosed cases in the province from 2019 to 2023. *P. vivax* was the predominant species, accounting for 78.7% (7940/10 094) of cases. Mixed infections comprised 1.5% (148/10 094) of cases, while 19.7% (1993/10 094) were *P. falciparum* and 0.1% (13/10 094) were *P. malariae*.

**Table 1:** Total number of types of malaria species identified in Kampong Speu Province, Cambodia, by year, 2019–2023

Malaria species/year	2019	2020	2021	2022	2023	Total
*P. vivax*	**3992 (73.0)**	**1982 (80.8)**	**1095 (90.0)**	**759 (93.2)**	**112 (88.9)**	**7940 (78.7)**
**Mixed^a^**	**135 (2.5)**	**9 (0.4)**	**–**	**4 (0.5)**	**–**	**148 (1.5)**
*P. falciparum*	**1357 (24.7)**	**462 (18.8)**	**122 (10.0)**	**47 (5.8)**	**5 (4.0)**	**1993 (19.7)**
*P. malariae*	**–**	**–**	**–**	**4 (0.5)**	**9 (7.1)**	**13 (0.1)**
**Total**	**5484 (100)**	**2453 (100)**	**1217 (100)**	**814 (100)**	**126 (100)**	10 094 (100)

Values are *n*(%); *P: Plasmodium*.

^a^ Mixed = *P. vivax* and *P. falciparum* coinfection.

### Profile of malaria cases with *P. vivax* or mixed infections

During the same period, a total of 8088 cases were identified with either *P. vivax* or mixed infections. The age of cases ranged from 1 to 83 years, with the majority (7069, 87.4%) falling within the 15–49-year age group. A significant portion of the overall cases (7253, 89.7%) were male, with a male-to-female ratio of 9:1. Among those with recorded mobile population status, the majority (3474/4892, 71.0%) were non-mobile (**Table 2**).

**Table 2:** Characteristics of malaria cases with *Plasmodium vivax* or mixed infections (*n* = 8088), Kampong Speu Province, 2019–2023

Characteristics	*n*(%)
**Age group (years)**	
** < 5**	**66 (0.8)**
**5–14**	**303 (3.7)**
15–49	7069 (87.4)
** ≥ 50**	**650 (8.1)**
**Sex**	
Male	**7253 (89.7)**
**Female**	**835 (10.3)**
**Mobility status**	
**Mobile**	**1418 (17.5)**
**Non-mobile**	**3474 (43.0)**
**Missing data^a^**	**3196 (39.5)**

^a^ Mobility status was not recorded for cases seen at health facilities.

Cases with severe illness were rare (44, 0.5%). The rest had simple or uncomplicated malaria. No deaths were reported. According to available surveillance data (2021–2023), 59.7% (1177/1970) of cases were classified as relapses based on a history of *P. vivax* infection in the past 12 months.

### G6PD testing and radical cure treatment

**Fig. 4:**
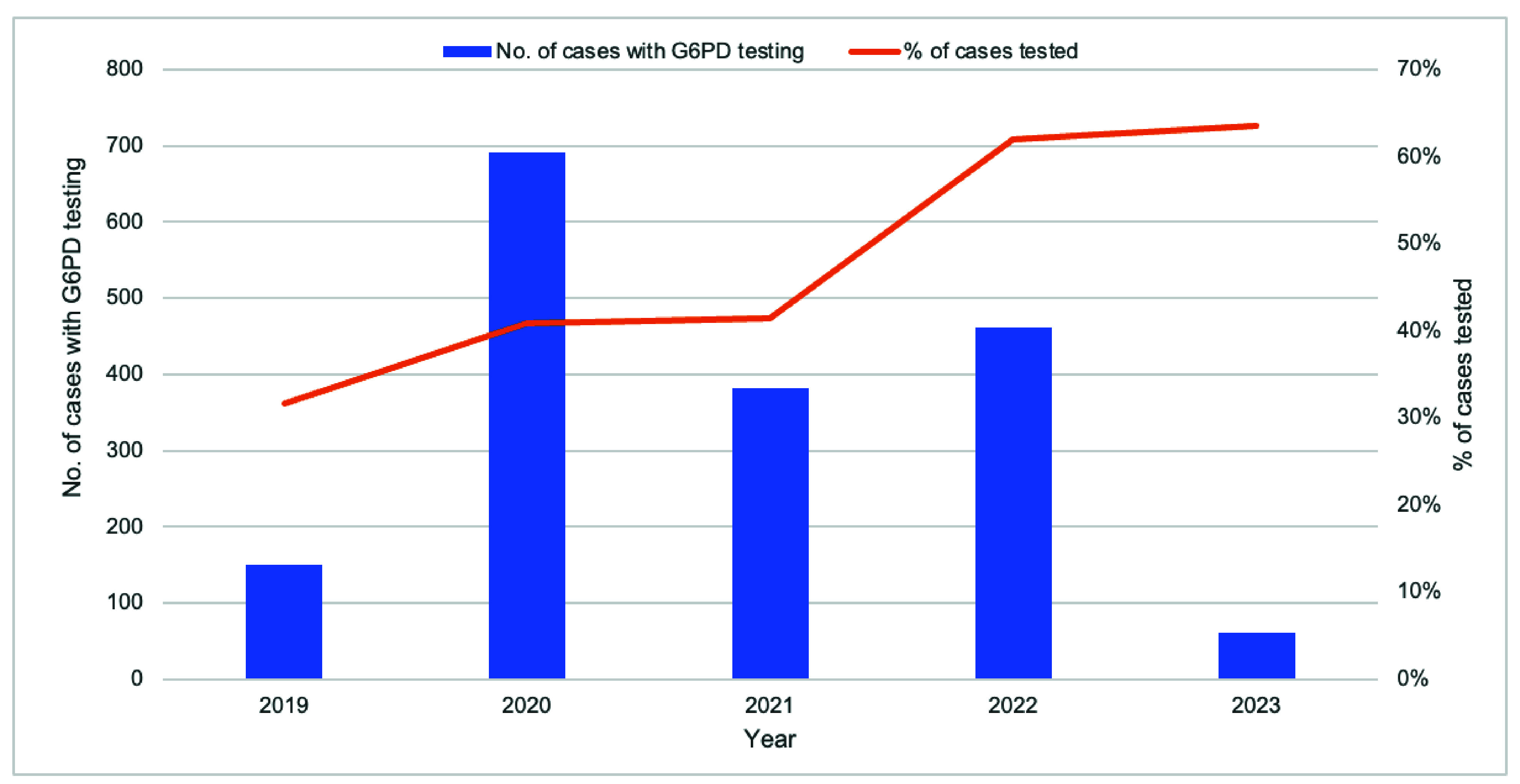
Number and percentage of eligible malaria cases tested for G6PD deficiency, Kampong Speu Province, Cambodia, 2019–2023

**Fig. 5:**
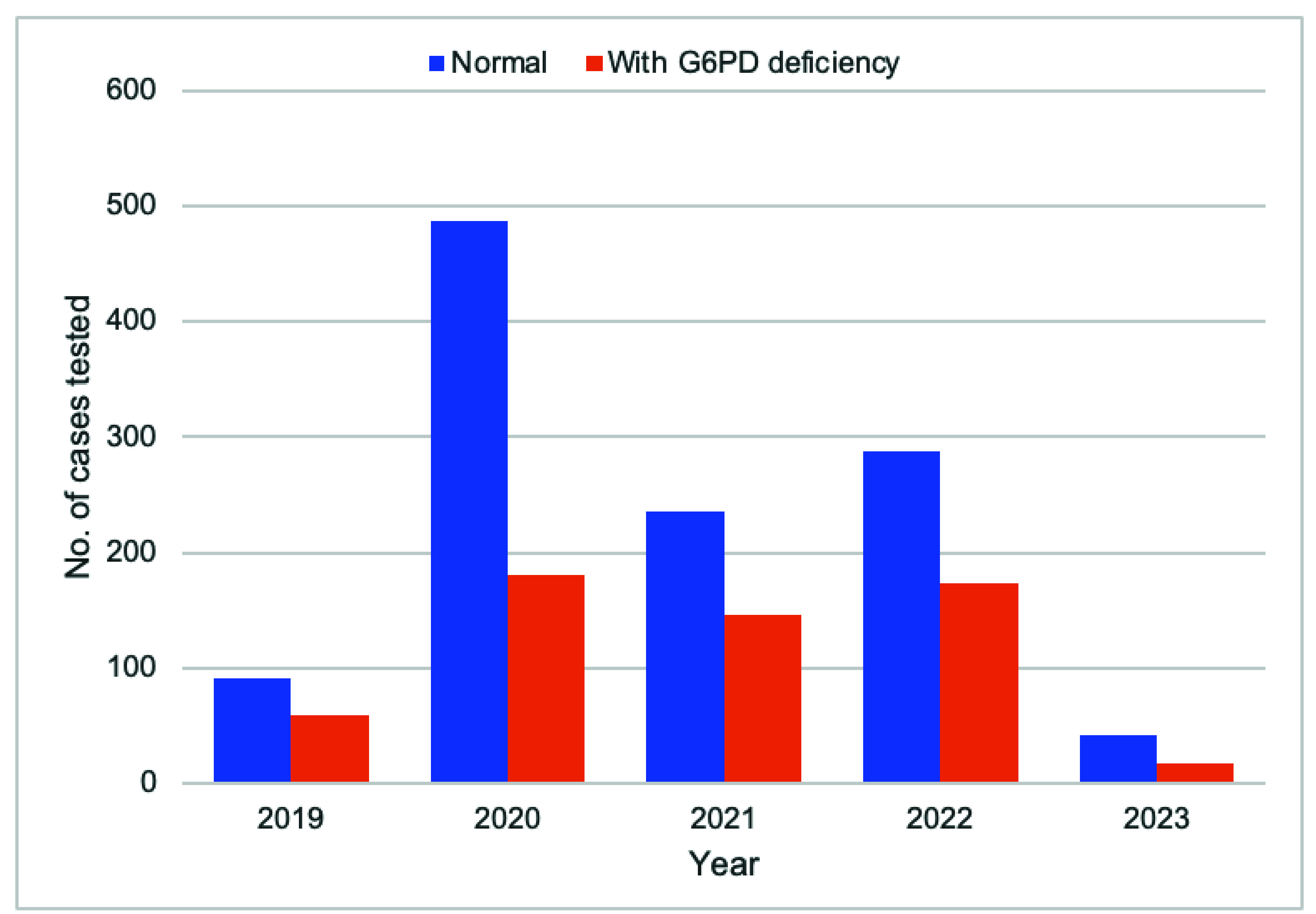
Number of G6PD deficiency test results compared to normal results by year, Kampong Speu Province, Cambodia, 2019–2023

**Fig. 6:**
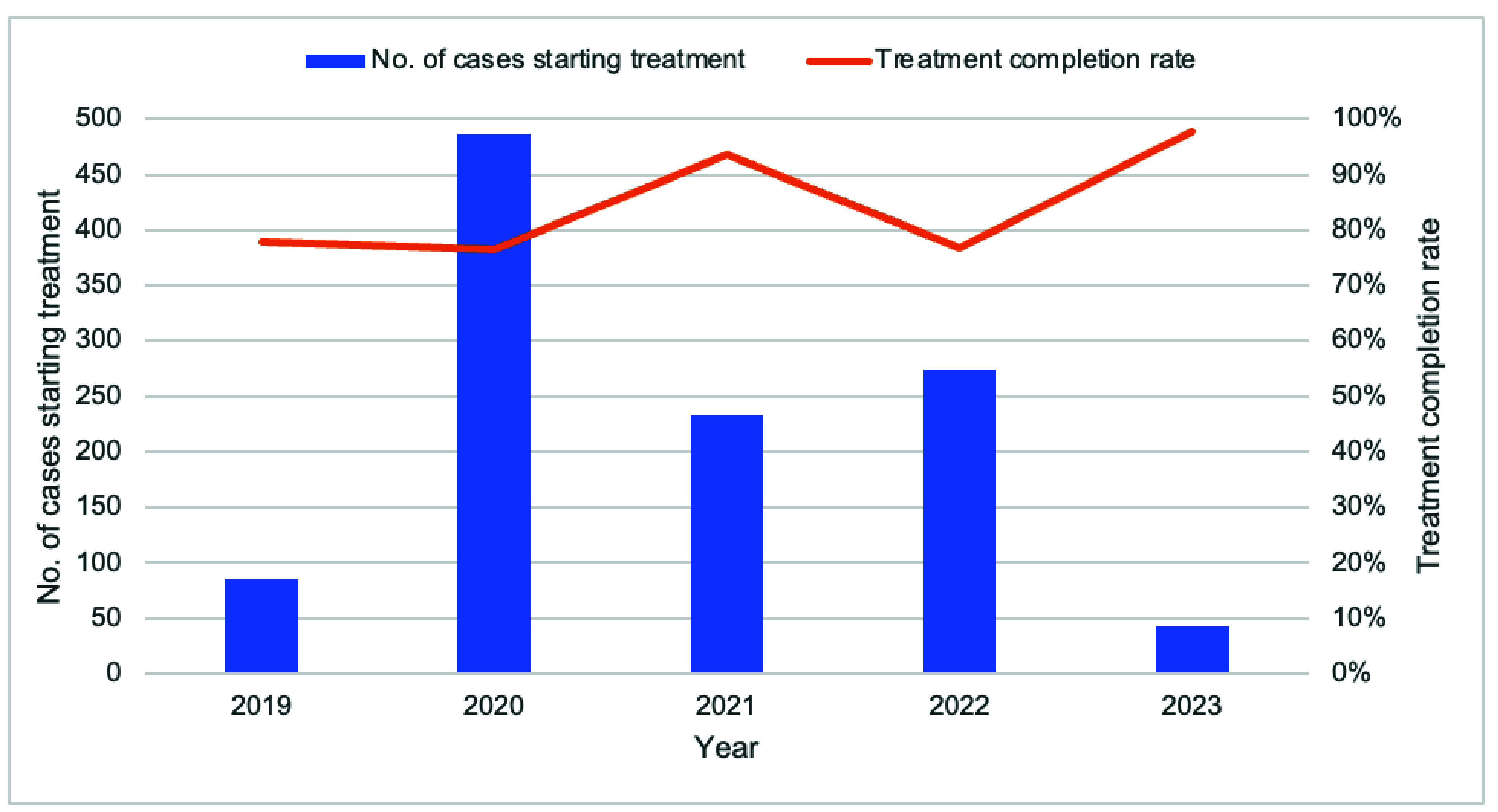
Number of cases starting radical cure of malaria species and treatment completion^a^ rates by year, Kampong Speu Province, Cambodia, 2019–2023

The proportion of eligible malaria cases tested for G6PD deficiency improved from 31.7% (151/476) in 2019 to 63.5% (61/96) in 2023 (**Fig. 4**). The majority (66.4%, 1144/1723) of those tested were not G6PD deficient, making them eligible for radical cure treatment (**Fig. 5**). The completion rate for radical cure treatment increased from 77.0% (67/87) in 2019 to 97.6% (41/42) in 2023 (**Fig. 6**).

[**insert** Figure 4]

[**insert** Figure 5]

[**insert** Figure 6]

### Annual parasite incidence

**Fig. 7:**
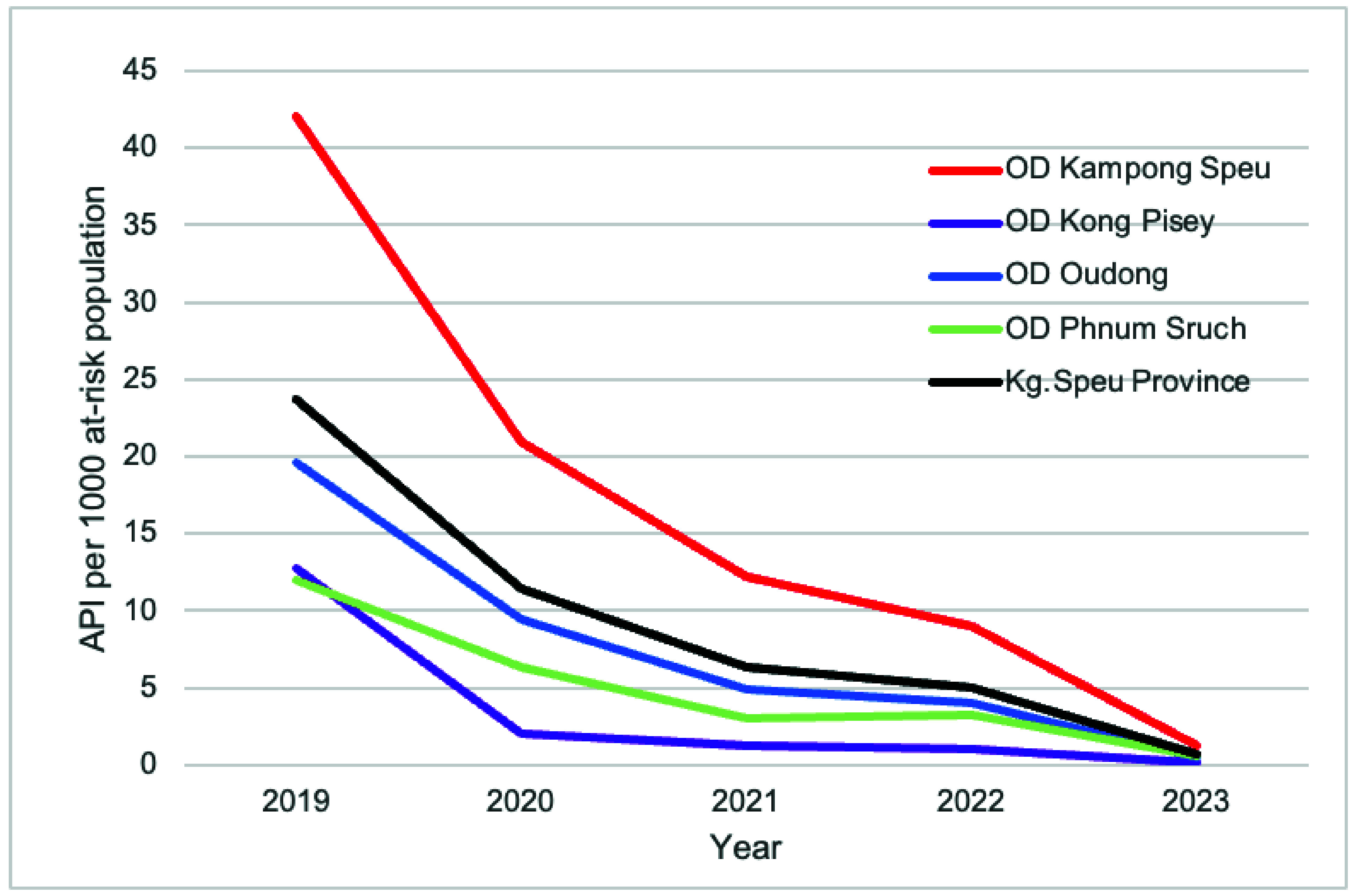
API^a^ by operational district, Kampong Speu Province, Cambodia, 2019–2023^b^

The API of *P. vivax* and mixed-infection cases demonstrated a substantial decrease from 23.8 per 1000 at-risk population in 2019 to 0.7 per 1000 in 2023. While all ODs in the province showed a decreasing API trend, Kampong Speu OD consistently recorded the highest (**Fig. 7**).

[**insert** Figure 7]

## Discussion

From 2019 to 2023, Kampong Speu Province experienced a consistent decline in malaria API, indicating steady progress towards eliminating malaria by 2025. This notable decrease in API can be attributed to several concerted efforts within the province. Key among these is the scaling up of active malaria screening, primarily carried out by VMWs who play an essential role in early detection and community-level interventions. Additionally, the increased completion rate of radical cure treatments among eligible cases has contributed significantly to this downward API trend by preventing relapse and by reducing transmission potential.

Despite the overall success, males aged 15–49 years continue to represent the most at-risk demographic for malaria in Kampong Speu Province. This aligns with findings from a comprehensive review of Cambodia’s malaria surveillance spanning 2006–2019, which identified a similar pattern. (*10*) The elevated risk among this group can be linked to socioeconomic and occupational factors, as Cambodian men frequently engage in work that requires them to travel and remain in forested areas for multiple days or weeks, such as logging, agriculture and construction. (*11*) Forested areas show higher exposure to the *Anopheles* mosquito vectors that thrive in such environments, thus increasing the risk of transmission.

Kampong Speu OD has consistently reported the highest API among the four ODs in the province. This could be attributed to its unique geographical and environmental characteristics. The presence of Mount Aoral, the tallest peak in Cambodia, contributes to extensive forest cover in the district. This forested terrain is a known habitat for malaria vectors and serves as a work location for many male residents. The combination of dense forest cover, high human activity within these areas, and limited access to malaria prevention and treatment in forested areas is mainly due to the difficult terrain and the mobile nature of seasonal work such as logging and foraging. Many people stay in the forest for extended periods, ranging from a few days to several weeks, and only seek care when symptoms appear, often after leaving, leading to delayed diagnosis and ongoing transmission. Mobile malaria workers offer some services, but coverage remains limited.

### Limitations

The data collected through the MIS are primarily derived from reports provided by health facilities and community-level health-care workers within Kampong Speu Province. However, this system does not include reports from military health services where malaria testing and treatment are also conducted. As a result, the overall number of reported malaria cases and the API presented in this study may be underestimated.

However, national malaria programme staff we spoke to confirmed that only seven cases of *P. vivax* were reported from the military camps in the province from 2019 to 2023 (personal communication to KL). Therefore, we believe that the trends observed in this report are representative of the malaria trends in the province.

### Conclusion

Kampong Speu Province has demonstrated considerable progress in its efforts towards malaria elimination. Continued momentum in achieving full malaria elimination will require enhanced and more focused interventions, particularly targeted at high-risk populations within Kampong Speu OD. Strengthening surveillance, improving access to preventive and curative services and engaging in strategies adapted to vulnerable groups will be crucial in ensuring that the province maintains and accelerates its path towards malaria elimination.

### Recommendations

Kampong Speu Province has shown significant progress towards eliminating malaria, but continued efforts are necessary to ensure sustainable results. Key focus areas for future interventions include:

**Implementing interventions that target high-risk populations.** Develop focused strategies to protect and serve populations at the highest risk of malaria, such as young adult males and forest-goers.
**Engaging with vulnerable groups.** Foster community engagement and education to raise awareness about malaria prevention, symptoms and treatment in collaboration with local organizations and health workers to reach underserved populations effectively.
**Strengthening surveillance.** Implement robust surveillance systems to track malaria cases in real time, enabling timely responses and resource allocation.
**Striving to meet the 1–3–7 target.** Ensure case notification within 1 day from diagnosis, reactive case detection within 3 days of notification of an index case, and foci investigation within 7 days of notification of a case with *P. falciparum* or an indigenous case regardless of the species.
**Improving access to services.** Determine whether the current prevention measures can be adapted and ensure that diagnostics and treatment are readily available and accessible in clinics, especially in remote areas. Continue to provide radical cure treatment to patients with *P. vivax* or mixed infections, including those with G6PD deficiency, in accordance with the latest treatment guidelines. (*8*)

By concentrating on these key strategies, Kampong Speu Province can build on its achievements and continue moving towards the goal of malaria elimination. Sustained commitment and innovation will be key to overcoming the remaining challenges.
